# Effect of Physiotherapeutic Rehabilitation on a Patient With an Iliac Fracture, and Superior and Inferior Pubic Rami Fracture With Foot Drop: A Case Report

**DOI:** 10.7759/cureus.33709

**Published:** 2023-01-12

**Authors:** Charul Dandale, Neha V Chitale, Pratik Phansopkar

**Affiliations:** 1 Musculoskeletal Physiotherapy, Ravi Nair Physiotherapy College, Datta Meghe Institute of Medical Sciences, Wardha, IND

**Keywords:** rehabilitation, physiotherapy, pubic rami, iliac wing fracture, foot drop

## Abstract

The sacroiliac joint is frequently broken apart by high-energy trauma, which increases fatalities and complications from pelvic injuries. Ilium fractures are high-energy pelvic fractures that often progress from the iliac crest to the greater sciatic notch. Concomitant head injury exsanguinations and uncontrollable bleeding within the pelvis are considered important causes of mortality. In contrast, some assume that such extensive bleeding is extremely uncommon and that accompanying injuries could result in increased mortality. A shorter healing period and faster patient mobilization are possible with surgically treated Tile's type B and C fractures. Accident-related fractures can lead to decreased independence and functioning, restricted mobility, lowered self-confidence, and a worse quality of life; these fractures are caused by trauma, most frequently from minor falls and age-related osteopenia. By reducing discomfort, restoring range of motion and muscular strength, and assisting with early ambulation/loading of the fractured limb, early physical therapy intervention speeds up the clinical recovery of patients with fractures.

When one cannot elevate the forefoot, it results in foot drop because of a lack of dorsiflexor strength in the foot. These may induce a risky antalgic gait, leading to falls-the diminished ability to lift the foot of the ankle or the toes (dorsiflexion). Injuries, including fractures, joint dislocations, or hip replacement surgery, can also result in a drop foot. The peroneal nerve, which innervates the tibialis anterior, is the muscle responsible for dorsiflexion, as it arises from the sciatic nerve's branch. Due to the foot drop, the anterior tibialis muscle will shorten and cause spasms in the calf muscle. After surgery, the patient was dependent and had difficulty going about his everyday life. However, the physiotherapy intervention improved the patient's pain and physical functionality. By lowering discomfort, restoring range of motion and muscular strength, and facilitating early ambulation/loading of the fractured limb, this study shows that combining definitive surgical methods with early physical therapy intervention speeds up the clinical recovery of patients with fractures.

## Introduction

Pelvic fractures are severe injuries linked to a variety of morbidities. Depending on the extent of the pelvic fractures, the amount of hemorrhage, and lesions to the brain, thorax, and abdomen, mortality rates can range from 10% to 50% [1]. Pelvic bone fractures are frequently seen in high-speed car accidents and constitute a medical emergency since they are complicated mainly by massive internal trauma, shock, and mortality. One of the deadliest and most debilitating injuries is complex pelvic fractures which are frequently not isolated [2]. Pelvic ring injuries are critical, potentially fatal wounds. An incidence of 23 per 100,000 per year has been recorded [3]. High-energy trauma frequently separates the sacroiliac joint, raising the risk of complications and fatalities in pelvic injuries. Iliac fractures, also known as high-energy pelvic fractures, usually extend from the iliac crest to the greater sciatic notch [4]. Intramedullary nailing, open reduction and internal fixation (ORIF), minimally invasive plate osteosynthesis (MIPO), and external fixation are some of the most popular surgical techniques [5].

Foot drop occurs when the forefoot cannot be lifted because the foot's dorsiflexion is weak. These may result in a risky antalgic gait, which could lead to falls [6]. It is characterized by significant weakness in the toe and ankle dorsiflexion. The foot and ankle dorsiflexor muscles regulate the foot's plantarflexion during heel strike and help the body clear the foot during the swing phase [7]. Due to the foot not clearing the floor, the foot drop causes a high stepping gait and increases the risk of falling. The toe drop is a medical term for the inability or weakened capacity to elevate the toes or raise the foot from the ankle (dorsiflexion). A slight knee flexion is seen in the high-stepping gait throughout the walk; this is done to keep the foot from being pulled to the ground. Dorsiflexion of the foot becomes challenging during the swing phase [8]. To maintain the foot's dorsiflexion, ankle-foot orthoses (AFOs) are widely used in mechanical foot drop treatments. The 'fixed' AFO device is restricted by its ability to hold the foot in a fixed flexion posture, whereas the 'dynamic' type has a bulkier appearance despite allowing for greater joint mobility. The main goals of physiotherapy are to improve proper function and decrease pain, adhesions, tightness, stiffness, and range of motion. Dislocation, bone loss, soft tissue injury, co-morbidities, and infections associated with numerous injuries may all have a negative impact on the treatment's prognosis. The objectives of physiotherapy include the treatment of fractures, promotion of healing, encouragement of weight-bearing, maintenance of weak muscles' strength, preservation of a joint range of motion, pain relief, and inflammation reduction. Few studies have been conducted in my area examining the crucial role that physiotherapy intervention plays in managing fractures and foot drops.

## Case presentation

A 53-year-old male was riding a bike and was severely injured in a vehicle accident and sent to the emergency department. He suffered a head injury, pelvis and left leg injuries, and several abdominal abrasions. Due to a fracture of his left leg's tibia and fibula immediately after the injury, he could not bear his weight on that lower limb. Then the patient was treated for a head injury and managed with plating on the left leg in a private hospital. He was then taken immediately to the hospital, where X-rays was done. The right-sided pelvic fracture was diagnosed by an orthopaedic surgeon along with a neuro deficit in the right foot drop. Then he underwent an operative procedure in which surgical correction of the right side sacroiliac joint and external fixator application for pelvis fracture was performed. The patient had a history of Diabetes Mellitus and Systemic Hypertension for three years on medications. The pain was sudden in onset and excruciating, aggravated by movement and relieved at rest. Intensity of pain was 9/10 on the numerical pain rating scale for lower limb motion and 5/10 for rest.

Timeline

The patient was admitted to the hospital on 03/09/2022. He was operated on for external fixator application for pelvis fracture and surgical correction of the right sacroiliac joint by CC screw on 16/09/2022. The physiotherapy intervention was started on 17/09/2022.

Findings and impression of investigations

**Figure 1:**
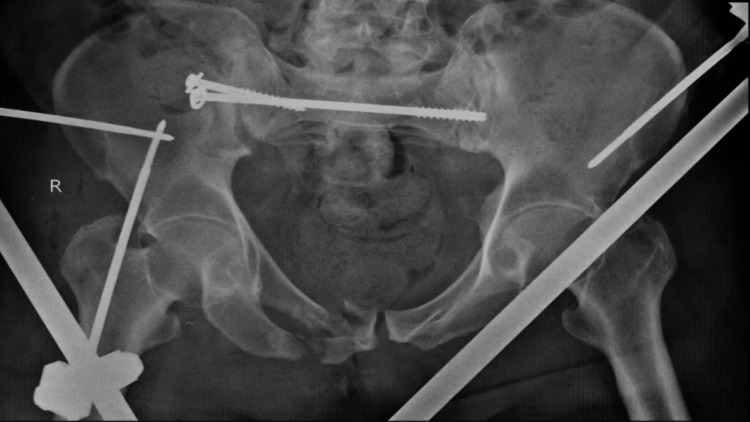
Postoperative x-ray showing the external fixator application for pelvis fracture and surgical correction of the right sacroiliac joint by CC screw. CC: Cannulated cancellous

X-ray results showed fractures in the right iliac wing and superior and inferior pubic rami. Left side sacrum involving ala and body with articular extension in the left side sacroiliac joint and left iliac bone. A communited minimally displaced fracture of the sacroiliac joint and the right iliac bone. Figure 1 shows the postoperative x-ray showing the external fixator application for pelvis fracture and surgical correction of the right sacroiliac joint by CC screw.

Diagnosis

The patient had a right-sided iliac wing fracture that extended toward the sacroiliac joint, a fracture of the sacral ala joint on the left side, and fractures of the superior and inferior pubic rami on the right side (lateral compression type II of Young and Burgess classification system).

Clinical findings

The patient gave his written, informed consent. The patient was conscious, cooperative, and well-oriented to the time, place, and person during the general assessment. The patient's hemodynamics was normal. On observation, ankle toe movements were present on the left side, ankle dorsiflexion of the right side could not be evaluated, and plantar flexion at the toes and ankle was elicited. A sensory examination on the right side revealed a significant reduction of light touch and pinprick in the right L4-L5 and S1 distributions. A foot drop assessment was performed, and shin and foot numbness was examined. The Stanmore assessment for foot drop evaluated grades as very poor, less than 55 points. The table mentioned below shows the range of motion (Table 1).

**Table 1:** The range of motion on day 1 of physiotherapy treatment.

Joint	Movement	Right		Left	
		Active	Passive	Active	Passive
Hip	Flexion	could not be assessed due to pain	0-30°	0-90^o^	0-100°
	Extension	Not assessed due to pain	0-15°	0-15°	0-20°
	Adduction	Not tested due to pain	0-30°	0-40°	0-50°
	Abduction	Not assessable due to pain	0-15°	0-25°	0-30°
Knee	Flexion	Not assessed because of pain	Not assessable because of pain	0-130°	0-130°
	Extension	Not assessed due to pain	Not tested due to pain	130-0°	130-0°
Ankle	Plantar Flexion	0-25°	0-50°	0-45°	0-50°
	Dorsi Flexion	0-5°	0-10°	0-10°	0-10°
	Inversion	0-10°	0-30°	0-30°	0-35°
	Eversion	0°	0-5°	0-10°	0-15°

Therapeutic intervention

Postoperative Management

Short-term goals included minimizing pain and swelling, improving joint range of motion, improving cardiovascular fitness, encouraging early mobilization, and preventing pressure/bed sores. Long-term goals were to re-educate patients to walk again, educate them in gait and balance, and increase their capacity for independent performance of activities of daily living (ADLs). Table 2 below contains physiotherapy interventions from week 1 to week 12.

**Table 2:** Physiotherapy interventions from week 1 to week 12 BD: Twice a day, SLR: Straight leg raise, ADLs: Activities of daily living, TD: Thrice a day

Sr. No	Goals	Intervention	Regimen
1.	Patient’s Education	To explain the value of physiotherapy to the patient and his family. To educate various positions for the patient, including a semi-fowlers position at first and 90-degree upright sitting afterwards.	The effects of exercise, ambulation, and position were explained while counselling the patient and caregivers. Pillow positioning was educated to the patient and their caregivers.
2.	Reducing Pain	Cryotherapy application at the fracture site.	Cryo packs four to five times daily for seven to eight minutes each from day 1 to week 1.
3.	Prevention of pulmonary complications & integumentary complications	Breathing Exercises: pursed lip breathing, deep breathing, spirometry. Ankle toe movements. Active range of motion of unaffected side. Hip and knee muscles static exercises.	One set of 10 repetitions with BD. Progression from week 2: 1 set of 15 repetitions with BD. Week 3& 4: 20 repetitions with 1 set and BD.
4.	Preventing denervation atrophy	Electrical stimulation at a frequency of 30-50 Hz. Anterior and lateral compartments were stimulated.	Twenty minutes a day.
5.	To maintain thigh muscle strength, improve range of motion, and decrease joint rigidity.	Active assisted straight leg raise (SLR) 0 – 5^o^. Progression of actively assisted SLR 0-15^o^ and heel slides 0-15^o^ in week 2. Active Straight Leg Raised 0-30^o^; SLR in the prone position 0-100^o^; abduction 0-100^o^; heel slides 0-30^o^; active-assisted dynamic quadriceps in sitting position 0-30^o ^progressed in weeks 3 and 4. Progression in week 5 to week 8: Exercises for the hips and knees that need self-resistance include resisting hip flexion, extension, and abduction while employing a weight cuff and a resistance band. For the gluteus maximus and medius, performing isometric exercises. SLR: 0-45^o^; SLR in prone 0-30^o^; abduction 0-15^o^; heel slides 0-45^o^; dynamic quadriceps 0-45^o^. SLR 0-60^o^; SLR in prone 0-20^o^; providing resistance; abduction 0-30^o^; heel slides 0-130^o^; dynamic quadriceps 0-70^o^ progressed in week 9 to week 12. Exercise requires resistance using the resistance band.	One set X 10 repetitions with BD in week 1 to week 4. Progress to 20 repetitions in 1 set TD in week 5 to week 8. Thirty repetitions in 1 set in week 8 to week 12.
6.	To improve and maintain hip abductor and adductor muscle strengthening and reduce joint rigidness.	Active assistive hip abduction 0-50^o^ and adduction 0-5^o^ in three days. Active hip abduction 0-10^o^ and adduction 10-0^o^ progressed in week 2.	1 set X 10 repetitions with BD in week 1 to week 4. Progress to 20 repetitions in 1 set TD in week 5 to week 8. 30 repetitions in 1 set in week 8 to week 12.
7.	To initiate tendoachilis stretching	Applying stretch to tendoachilis will maintain the normal flexibility of dorsiflexion. Progressed in week 4 and continued till week 12.	Thirty seconds hold with three sets
8.	Initiate the trunk mobility and hip mobility	Unilateral bridging along with assistance of the unaffected leg beginning on the fifth post-operative day in week 1.	Ten repetitions with 1 set BD.
9.	Initiate weight bearing	Partial-weight bearing combined with pivot shifting. The patient shifted from prolonged sitting to upright sitting between weeks 3 and 4, followed by standing re-education and eventually walking re-education. Standing and walking re-education progressed in week 6.	Initiated between weeks 3 and 4
10.	Focusing on ADLs	During week 4, using the western toilet, dressing, undressing, and personal hygiene. In week 9-week 12, independent in ADLs.	Starting from week 3
11.	Gait Training	Spot marching, jumping hurdles, climbing stairs, walking backwards, walking in tandem, and jumping high steps and obstacles were all encouraged.	Starting from week 9

Follow-up and outcomes

After the physiotherapy rehabilitation, the patient was able to perform normal activities without experiencing any pain or decrease in range of motion. The patient noted an increased range of motion (ROM) and muscle strength (Table 3); the Stanmore assessment score was also increased from poor to very good grade, which is 100-85 points. Table 4 shows the range of motion evaluation after physiotherapy treatment.

**Table 3:** The method of evaluating muscle strength is the Oxford Scale (aka Medical Research Council Manual Muscle Testing scale) pre and post-physiotherapy management.

Muscle Group	Pre Physiotherapy		Post Physiotherapy	
	Right	Left	Right	Left
Hip Flexors	Not tested due to pain.	Not tested due to pain.	3	5
Hip Extensors	Not tested due to pain.	Not tested due to pain.	3	4
Hip Abductors	Not tested due to pain.	Not tested due to pain.	4	4
Hip Adductors	Not tested due to pain.	Not tested due to pain.	4	4
Knee Flexors	Not tested due to pain.	Not tested due to pain.	3	4
Knee Extensors	Not tested due to pain.	Not tested due to pain.	3	4
Ankle Plantar Flexors	3	4	4	5
Ankle Dorsiflexors	1	4	4	5

**Table 4:** The range of motion evaluation after the physiotherapy treatment. NA: Not accessible

Joint	Movement	Right		Left	
		Active	Passive	Active	Passive
Hip	Flexion	NA	0-35°	0-110°	0-120°
	Extension	NA	0-15°	0-15°	0-20°
	Adduction	NA	0-20°	0-40°	0-50°
	Abduction	NA	0-15°	0-25°	0-30°
Knee	Flexion	NA	NA	0-130°	0-130°
	Extension	NA	NA	130-0°	130-0°
Ankle	Plantar Flexion	0-25°	0-50°	0-45°	0-50°
	Dorsi Flexion	0-5°	0-10°	0-10°	0-10°
	Inversion	0-15°	0-35°	0-30°	0-35°
	Eversion	0-10°	0-15°	0-10°	0-15°

## Discussion

The patient, in this case, was unable to lift his forefoot due to right hip pain, edema, a restricted range of motion, reduced strength, and right-side dorsiflexor weakness. After the patient was evaluated, a therapeutic plan was created to address each symptom. This program comprised both active and passive ROM exercises, muscle strengthening, and the utilization of a variety of modalities.

Pelvic ring fractures and acetabulum fractures generated by high-energy trauma are frequently displaced to the point that surgery is required. This is because the common injury mechanism leading to these fractures-motor vehicle collisions-impacts several other connected injuries [2]. However, 31% of the individuals used walking aids, the majority of which were canes, as a safety precaution. Many older people also require assistance in some form. To compare how hip fractures are treated, a uniform approach emphasizing early weight bearing and mobility is necessary. In certain circumstances, such as in elite athletes and those with significantly displaced fractures, surgical intervention may be required in order to minimize healing time [4]. Foot drop occurs when the dorsiflexors of the foot are not strong enough to lift the forefoot [9]. The dorsiflexors of the foot and ankle weaken, resulting in an equinovarus deformity. Steppage gait is the tendency to walk with exaggerated knee and hip flexion to maintain one's toes off the floor during the swing phase [6]. Co-morbidities like diabetes mellitus and hypertension may delay the healing process, but with proper medications and precautions, patient improvement is observed. Few studies have been conducted in our area examining the crucial part that rehabilitation interventions play in managing fractures and foot drop.

A device attached externally to the limb helps with function, gait stabilization, pain management through load distribution, correction of flexible abnormalities, and slowing the evolution of fixed deformities. Such as an ankle-foot orthosis (AFO). These typically include a shoe insert, calf shell, and calf strap and can be flexible or rigid. Throughout the intervention, the patient received the necessary education about what to expect, possible issues, how to handle them, and the next steps after the surgery. For the patient to be more capable of managing postoperative issues, their psychological condition must improve. The adaptive postoperative prognosis is improved by early, unrestricted mobilization and complete weight-bearing with physiotherapy [10]. Prevent further problems, including contractures, bed sores, and deep vein thrombosis.

## Conclusions

Physical function, independence, and everyday living abilities are improved with physiotherapy. Muscle strength from 0 to 5, range of motion from minimal to maximum, and sensations and activities of daily living are maintained and improved with physiotherapy rehabilitation and week-wise progression in treatment. The patient's determination to continue receiving appropriate care and physical therapy contributed to his positive progress. By lowering pain, improving range of motion and muscular strength, and promoting early ambulation, this study shows how combined definitive surgical treatments with early physiotherapy therapy helps to enhance the clinical improvement in fracture patients.
